# Detecting oral cancer in edentulous patients during denture fabrication by Prosthodontists

**DOI:** 10.6026/973206300210997

**Published:** 2025-05-31

**Authors:** Pushkar Gupta, Pravin M. Parmar, Litto Manual, Trapti Jaiswal, Angela Justin, Nitin Purohit, Radhe Shyam Singh, Saurabh Gupta

**Affiliations:** 1 Department of Prosthodontics and Crown & Bridge, Hitkarini Dental College and Hospital, Jabalpur, Madhya Pradesh, India; 2 Prosthodontist, Gujarat University, General Hospital Botad, Gujarat, India; 3 Department of Prosthodontics, Mar Baselios Dental College, Kothamangalam, Kerala-686691, India; 4 Department of Prosthodontics and Crown & Bridge, Index Institute of Dental Sciences, Indore, Madhya Pradesh, India; 5 Department of Prosthodontics and Crown & Bridge, Hitkarini Dental College and Hospital, Jabalpur, Madhya Pradesh India; 6 Department of Dentistry, District Hospital, Rudraprayag, Uttarakhand, India; 7 Intern, Kalinga Institute of Dental Sciences, Kalinga Institute of Industrial Technology (KIIT), Deemed to be university, Bhubaneswar, Odisha, India; 8 Consultant, Periodontist and Implantologist, Jammu and Kashmir, India

**Keywords:** Oral cancer, Prosthodontists, edentulous, denture fabrication, potentially malignant disorders (PMDs), tobacco, oral screening

## Abstract

Detection of potentially malignant disorders (PMDs) and oral cancer among completely edentulous patients by Prosthodontists is of interest. Hence, a thorough soft tissue examination was conducted at five key stages of denture fabrication on 500 edentulous patients. Information on tobacco and alcohol consumption was collected and biopsies were performed. Of the 500 participants, 124 (24.8%) presented with mucosal lesions, and 68 (13.6%) were confirmed as PMDs through histological analysis. There was a significant correlation between lesion occurrence and the use of tobacco (p < 0.001) and alcohol (p = 0.02). Most lesions were identified during the final impression and try-in appointments. Thus, prosthodontists hold a crucial position in the early detection of oral cancer.

## Background:

Oral cancer remains one of the most prevalent malignancies in India, contributing substantially to cancer-related morbidity and mortality. The risk is notably higher in the elderly, particularly among those who are completely edentulous [1]. Loss of teeth often leads to reduced engagement with routine dental care, resulting in delayed diagnosis of oral health issues, including potentially malignant disorders (PMDs) and oral cancer. This lack of regular dental surveillance in edentulous patients poses a serious challenge in early detection and timely intervention [2, 3]. Prosthodontists, however, play a unique and essential role in this context. As specialists responsible for the rehabilitation of edentulous patients through complete denture fabrication, they engage with patients across multiple clinical stages. These stages-ranging from initial examination to final denture delivery-involve repeated and detailed evaluation of the oral cavity. This interaction presents a valuable opportunity for Prosthodontists to observe, identify, and monitor abnormal mucosal changes that may otherwise go unnoticed [4, 5]. Given the widespread prevalence of risk factors such as tobacco use and alcohol consumption in India, especially in older populations, the role of Prosthodontists extends beyond prosthetic rehabilitation to that of an important contributor in the early detection of PMDs and oral malignancies [6]. Their clinical vigilance and timely referrals can significantly improve patient outcomes by facilitating early diagnosis and management. Therefore, it is of interest to explore the potential role of Prosthodontists as frontline healthcare providers in the early identification of oral cancer and PMDs among edentulous patients during the denture fabrication process.

## Materials and Methods:

The present study employed a clinical cross-sectional correlation design and included a total of 500 completely edentulous patients. Participants were selected based on specific inclusion criteria: individuals aged 40 years or older, completely edentulous, and willing to undergo a biopsy if clinically indicated. Patients with a prior history of treated oral malignancy or those with systemic immunosuppression were excluded from the study to avoid potential confounding factors. Data collection was carried out using a structured questionnaire, which was completed during the clinical examination stages of denture fabrication. The questionnaire was designed in a tabular format and comprised several key sections (Table 1). The demographic section recorded age, gender, socioeconomic status, level of education, and whether the participant resided in an urban or rural area. The habit section captured information on the use of tobacco-both smoking and smokeless forms-as well as alcohol consumption, including the duration of these habits. Details regarding oral lesions included their site, size, color, surface texture, and associated symptoms such as pain or burning sensations. The clinical stage of detection section noted the point during the denture fabrication process at which any mucosal abnormality was first observed. These stages included primary impression, final impression, jaw relation, try-in, and denture delivery. Lastly, the referral and histopathology section documented whether the patient was referred for further evaluation and biopsy, along with the final histopathological diagnosis, if applicable. This structured approach enabled a systematic assessment of potential risk factors and the clinical presentation of lesions, while also evaluating the specific stages during which Prosthodontists are most likely to detect suspicious mucosal changes.

## Data collection tool:

Structured questionnaire filled during clinical examination

## Results:

A total of 500 completely edentulous patients participated in the study, with a male predominance (64%) and the remaining 36% being female. The majority of participants (58%) reported tobacco use, while 30% reported alcohol consumption. Urban residents made up 55% of the sample, with the remaining 45% from rural areas, as shown in Table 2. Out of the 500 patients, 124 individuals (24.8%) exhibited visible mucosal lesions during clinical examination. These lesions were identified at various stages of denture fabrication. The highest detection rate was observed during the try-in stage (30.6%), followed by the final impression stage (27.4%). Fewer lesions were detected during jaw relation (16.1%), denture delivery (16.1%), and primary impression (9.7%) stages (Table 3), highlighting the importance of thorough soft tissue evaluation throughout all clinical steps. A detailed examination of lesion characteristics revealed that the most commonly affected sites included the buccal mucosa, alveolar ridge, and soft palate. Lesions varied in size, with most ranging from 0.5 to 2 cm. In terms of color, lesions were predominantly white (leukoplakia-like), red (erythroplakia-like), or mixed red and white. Texture ranged from smooth to rough or corrugated surfaces. Common symptoms included mild pain and burning sensation, especially in lesions associated with oral submucous fibrosis and lichen planus (Table 4). Histopathological analysis confirmed the presence of potentially malignant disorders (PMDs) and malignancies in several cases. Of the 124 biopsied lesions, Leukoplakia was the most common diagnosis (25.8%), followed by Erythroplakia (14.5%), Oral Submucous Fibrosis (8.1%), and Lichen Planus (6.5%). Squamous Cell Carcinoma (SCC) was confirmed in 4% of cases, while 41.1% of lesions were classified as benign or non-specific (Table 4). Statistical analysis revealed a significant association between tobacco use and the presence of mucosal lesions (p < 0.001), with 82.2% of lesion-positive patients being tobacco users. Similarly, alcohol use was significantly associated with lesion occurrence (p = 0.02), with 41.9% of lesion-positive patients reporting alcohol consumption. These associations were analyzed using the Chi-square test, and all data were processed using SPSS version 25, with a p-value of <0.05 considered statistically significant (Table 5).

## Discussion:

Oral cancer remains a significant public health concern in India, particularly among edentulous individuals who often miss regular dental screenings. The absence of teeth can mask early mucosal changes, delaying diagnosis [7]. Prosthodontists, through multiple denture fabrication visits, are uniquely positioned to detect potentially malignant disorders (PMDs). Early identification during these routine appointments can play a crucial role in improving patient outcomes [8, 9]. In the present study, the majority of participants were male (64%), which is consistent with findings by Singh *et al.* (2021), who reported a male predominance in oral potentially malignant disorder (PMD) cases, attributing it to higher tobacco and alcohol use among males in rural and semi-urban Indian populations [9]. Similarly, Pandya *et al.* (2020) observed a significant male-to-female ratio in oral cancer cases, underlining gender-related risk exposure and health-seeking behavior disparities [10]. Tobacco use was noted in 58% of patients, aligning closely with the Global Adult Tobacco Survey (GATS) India 2016-17, which reported 57% of adult males in India consume some form of tobacco [11]. This supports the strong correlation between tobacco habits and mucosal lesion development in edentulous patients. Alcohol use was reported in 30% of participants, which is somewhat lower compared to urban prevalence rates but consistent with Sankaranarayanan *et al.* (2001), who found alcohol to be a co-factor in oral cancer progression, especially when combined with tobacco [12]. The urban-rural distribution in this study showed 55% urban and 45% rural participation, highlighting a relatively balanced representation. However, rural populations often have less access to preventive care and higher tobacco consumption, as also noted in Sinha *et al.* (2012), where rural groups showed delayed oral cancer diagnosis due to limited dental awareness and accessibility [13]. In this study, the highest number of mucosal lesions in completely edentulous patients were detected during the try-in stage (30.6%), followed closely by the final impression stage (27.4%). These findings highlight the critical role of repeated clinical contact during prosthodontic appointments, particularly in later stages when soft tissues are more exposed and visible during procedural steps like border molding and occlusal trial. The relatively lower detection at the primary impression stage (9.7%) may be attributed to a lack of thorough tissue inspection during preliminary appointments, often focused more on anatomical landmarks than mucosal pathology. The jaw relation and denture delivery stages also presented detection opportunities, each accounting for 16.1% of the cases. These trends are comparable to Alqutaibi *et al.* (2021), observed a response rate of 57.2% (N=143). A significant proportion of respondents (79%) indicated that they routinely screen all new patients for mucosal abnormalities. However, only 58% continued this practice during recall appointments, and 61.5% reported offering advice on tobacco or smoking cessation. When it came to experience with potentially malignant lesions, 79.7% had identified a suspicious lesion at some point, and 83.2% had referred such patients for further evaluation. While 65% of prosthodontists felt confident in their ability to independently detect oral cancer, only 40% believed they could effectively motivate patients to quit smoking. The most commonly cited obstacles to oral cancer screening included insufficient training, lack of confidence, limited time, and inadequate financial incentives [14]. The findings support the integration of routine soft tissue examination protocols in prosthodontic practices, particularly during final impression and try-in visits, when visibility and tissue manipulation are optimal. The buccal mucosa emerged as the most frequently affected site (45.2%), aligning with common sites of tobacco quid placement, particularly among smokeless tobacco users-a trend supported by Patil *et al.* (2015) [15], who identified the buccal mucosa as the primary location for leukoplakia and oral submucous fibrosis in Indian populations. Most lesions were between 1-2 cm in size (61.3%), and the predominant colors observed were white (41.9%) and mixed red-white (38.7%), suggestive of leukoplakic and potentially malignant lesions. Rough textures (43.5%) and symptoms such as mild pain (58.1%) and burning sensation (46.8%) were frequently noted, particularly in lesions diagnosed as oral submucous fibrosis or lichen planus. Histopathologically, leukoplakia (25.8%) was the most common diagnosis, followed by erythroplakia (14.5%), consistent with the findings of Warnakulasuriya *et al.* (2007), who emphasized the prevalence of these lesions in high-risk populations and their potential for malignant transformation [16]. The present study revealed a strong association between tobacco use and the presence of mucosal lesions, with 82.2% of lesion-positive individuals reporting tobacco consumption (p < 0.001). This finding is in concordance with Gupta *et al.* (2016) [17], who documented tobacco as a significant etiological factor in the development of potentially malignant disorders (PMDs), particularly in edentulous and high-risk populations. Similarly, alcohol use was significantly associated with lesion occurrence (41.9% vs. 26%, p = 0.02), reflecting the synergistic effect of alcohol and tobacco in mucosal carcinogenesis, as supported by studies such as by Blot *et al.* (1988) [18]. These associations underscore the necessity for detailed habit history during prosthodontic rehabilitation, reinforcing the role of prosthodontists in early detection and referral of suspicious lesions. This study was limited to a single-center cross-sectional design, which may affect the generalizability of results to broader populations. Additionally, patient self-reporting of habits may introduce recall bias. Future multicentric longitudinal studies are recommended to track lesion progression and validate early detection protocols. Integrating routine mucosal screening into prosthodontic practice, along with habit cessation counseling, can significantly improve early diagnosis rates. Advancements in diagnostic tools such as auto-fluorescence and AI-based lesion detection may further enhance clinical vigilance in the future.

## Conclusion:

Denture fabrication presents a valuable opportunity for oral cancer screening. Data shows that there is a significant correlation between lesion occurrence and the use of tobacco and alcohol. Most lesions were identified during the final impression and try-in appointments. Hence, Prosthodontists should be trained and mandated to conduct thorough mucosal assessments, improving early diagnosis rates, especially in high-risk populations.

## Figures and Tables

**Table 1:** Questionnaire

**Section**	**Parameters**
Demographics	Age, Gender, SES, Education, Urban/Rural
Habits	Tobacco (Smoking/Smokeless), Alcohol, Duration
Lesion details	Site, Size, Color, Texture, Symptoms (pain, burning)
Clinical stage of detection	Primary impression, final impression, jaw relation, try-in, denture delivery
Referral & histopathology	Yes/No, final diagnosis

**Table 2:** Demographic and habit profile (n=500)

**Variable**	**Category**	**Frequency**	**Percentage**
Gender	Male	320	64%
	Female	180	36%
Tobacco Use	Yes	290	58%
	No	210	42%
Alcohol Use	Yes	150	30%
	No	350	70%
Urban		275	55%
Rural		225	45%

**Table 3:** Lesion detection by stage

**Stage**	**Lesions Detected (n=124)**	**Percentage (%)**
Primary impression	12	9.70%
Final impression	34	27.40%
Jaw relation	20	16.10%
Try-in	38	30.60%
Denture delivery	20	16.10%

**Table 4:** Lesion characteristics and histopathological diagnosis (n = 124)

**Parameter**	**Category**	**Frequency (n)**	**Percentage (%)**
Site of Lesion	Buccal mucosa	56	45.20%
	Alveolar ridge	38	30.60%
	Soft palate	30	24.20%
Size of Lesion	0.5 - 1 cm	48	38.70%
	1 - 2 cm	76	61.30%
Color	White (Leukoplakia-like)	52	41.90%
	Red (Erythroplakia-like)	24	19.40%
	Mixed red and white	48	38.70%
Texture	Smooth	36	29.00%
	Rough	54	43.50%
	Corrugated	34	27.40%
Symptoms	Mild pain	72	58.10%
	Burning sensation	58	46.80%
Histopathological Diagnosis	Leukoplakia	32	25.80%
	Erythroplakia	18	14.50%
	Oral Submucous Fibrosis	10	8.10%
	Lichen Planus	8	6.50%
	Squamous Cell Carcinoma (SCC)	5	4.00%
	Benign/Non-specific findings	51	41.10%

**Table 5:** Association between tobacco, alcohol use and the presence of mucosal lesions

**Variable**	**Lesions Present (n=124)**	**Lesions Absent (n=376)**	**p-value**
Tobacco use	102 (82.2%)	188 (50%)	<0.001
Alcohol use	52 (41.9%)	98 (26%)	0.02
